# Fur-regulated MntH mediates metal homeostasis and immune evasion in *Yersinia pseudotuberculosis*

**DOI:** 10.1007/s44154-026-00348-8

**Published:** 2026-11-11

**Authors:** Peishuai Fu, Fengge Song, Nuoping Xu, Jitong Jiao, Liya Zhang, Xiaofang Ma, Danyang Yu, Yuqi Liu, Yuxin Zuo, Yantao Yang, Zhiyan Wei, Yao Wang, Xihui Shen, Changfu Li, Lingfang Zhu

**Affiliations:** Shaanxi Key Laboratory of Agricultural and Environmental Microbiology, College of Life Sciences, Northwest A&F University, Yangling, Shaanxi 712100 P. R. China

**Keywords:** Calprotectin, Iron acquisition, Manganese acquisition, Nutritional immunity, Virulence

## Abstract

During infection, host nutritional immunity restricts bacterial access to essential transition metals, in part through calprotectin (CP), a host metal-binding protein that sequesters manganese (Mn) and iron (Fe). However, whether the MntH transporter enables *Yersinia pseudotuberculosis* (*Yptb*) strain YPIII to withstand this CP-mediated metal restriction remains unclear. To address this question, we characterized the regulation and function of MntH in *Yptb* YPIII using molecular, genetic, physiological, and mouse-infection approaches. We found that MntH is a conserved member of the natural resistance-associated macrophage protein (Nramp) family whose expression is directly repressed by the ferric uptake regulator (Fur). Deletion of *mntH* reduced intracellular Mn and Fe accumulation, impaired bacterial growth under metal-limited conditions, and increased sensitivity to peroxide- and hydroxyl radical (HR)-associated stresses. In vitro, deletion of *mntH* impaired bacterial growth under CP-imposed metal restriction, and this defect was rescued by supplementation with Mn or Fe. In orally infected mice, the Δ*mntH* mutant yielded lower bacterial burdens in the feces and cecum, reduced competitive fitness, and milder cecal inflammation and liver damage than the wild-type strain. Notably, *Yptb* infection elevated cecal CP expression and depleted fecal Mn and Fe, whereas *mntH* expression was induced during infection, supporting a role for MntH in countering host-imposed metal limitation. Collectively, these findings establish Fur-regulated MntH as a key determinant of metal homeostasis, stress tolerance, and intestinal fitness in *Yptb* and provide insight into how this pathogen adapts to host nutritional limitation.

**Supplementary Information:**

The online version contains supplementary material available at https://doi.org/10.1007/s44154-026-00348-8.

## Introduction

Metal ions play a vital role in the survival and virulence of bacteria (Murdoch and Skaar 2022). Among these, manganese supports oxidative stress resistance and central metabolism, whereas iron functions as a redox-active cofactor in multiple cellular and virulence-associated processes (Bosma et al. 2021; Martin and Waters 2022; Sepúlveda Cisternas et al. 2018; Wang et al. 2009). To secure these vital resources, bacteria have evolved multiple sophisticated transport systems, encompassing high-affinity ATP-binding cassette transporters, siderophore-mediated uptake pathways, and the natural resistance-associated macrophage protein (Nramp) family transporters (Hood and Skaar 2012). Within the Nramp family, MntH emerges as a conserved manganese transporter, facilitating Mn uptake from the environment to sustain bacterial functions and enhance bacterial survival under stress conditions (Bozzi et al. 2016; Makui et al. 2000; Perry et al. 2012; Champion et al. 2011; Wang et al. 2025).

Although metal ions are important cofactors in numerous cellular processes, their accumulation beyond physiological levels can be cytotoxic (Chandrangsu et al. 2017). Therefore, metal acquisition systems are tightly regulated to balance nutritional demands and prevent metal toxicity (Murdoch and Skaar 2022). For example, in *Yersinia pestis*, the ferric uptake regulator (Fur) senses intracellular iron concentrations and represses the expression of iron acquisition-related genes under iron-replete conditions (Gao et al. 2008). Beyond iron homeostasis, the uptake of other essential metals like manganese is similarly regulated by specialized transcription factors (Que and Helmann 2000; Hood et al. 2017; Zuo et al. 2023). For instance, manganese uptake is regulated by the metal-specific transcriptional regulator MntR in *Bacillus subtilis* (Que and Helmann 2000), and by the manganese uptake regulator Mur in *Rhizobium leguminosarum* (Hood et al. 2017). Additionally, *Yersinia pseudotuberculosis* (*Yptb*) relies on Fur to maintain metal ion homeostasis and mitigate potential toxicity (Zuo et al. 2023). These complex regulatory networks highlight how bacteria precisely control metal homeostasis to adapt to varying environmental conditions, ensuring survival and virulence.

To restrict microbial access to essential metals during infection, hosts have evolved nutritional immunity, a critical defense strategy characterized by the sequestration of metal ions through high-affinity binding proteins, such as transferrin, lactoferrin, and calprotectin (CP) (Hood and Skaar 2012). CP, a heterodimeric complex of S100A8 and S100A9 proteins, exerts potent antimicrobial activity by chelating manganese (Mn^2+^), iron (Fe^2+^), and zinc (Zn^2+^), thereby limiting their availability to invading pathogens (Corbin et al. 2008; Kehl-Fie et al. 2011; Nakashige et al. 2015; Zygiel and Nolan 2018). In response, several pathogens, including *Salmonella enterica* serovar Typhimurium and *Staphylococcus aureus*, use conserved manganese transporters such as MntH, SitABCD, or MntABC to resist CP-mediated manganese sequestration (Diaz-Ochoa et al. 2016; Kehl-Fie et al. 2013).

*Yptb*, an enteric Gram-negative pathogen widely distributed in the environment, uses specialized transport systems to acquire manganese, zinc, and iron, which are not only essential for bacterial growth but also important in competing against host immune defenses (Zhu et al. 2021; Liu et al. 2023; Dai et al. 2026). For instance, TssS, a type VI secretion system (T6SS) effector, promotes manganese acquisition and interferes with host immunity (Zhu et al. 2021); YezP facilitates zinc acquisition and modulates host immune responses (Liu et al. 2023). More recently, SfeP was identified as a T6SS-secreted Fe-binding effector that enables *Yptb* to counter CP-mediated Fe restriction during infection (Dai et al. 2026). These systems reflect a broader strategy by *Yptb* to overcome host nutritional immunity, enhance colonization, and ensure persistence within the host. Despite the recognized importance of these metal acquisition pathways, how *Yptb* coordinates the expression and function of multiple transporters and regulators in response to dynamic environmental cues remains poorly understood.

In this study, we identified MntH as a Fur-regulated metal transporter in *Yptb* YPIII. Our results show that MntH contributes to manganese and iron acquisition, supports growth under calprotectin-imposed metal limitation, and promotes stress tolerance and gut fitness during infection. These results highlight the critical role of MntH and its regulatory mechanisms in bacterial adaptation to host-imposed nutritional immunity.

## Results

### Identification of a conserved MntH transporter in the *Yptb* YPIII strain

**Fig. 1:**
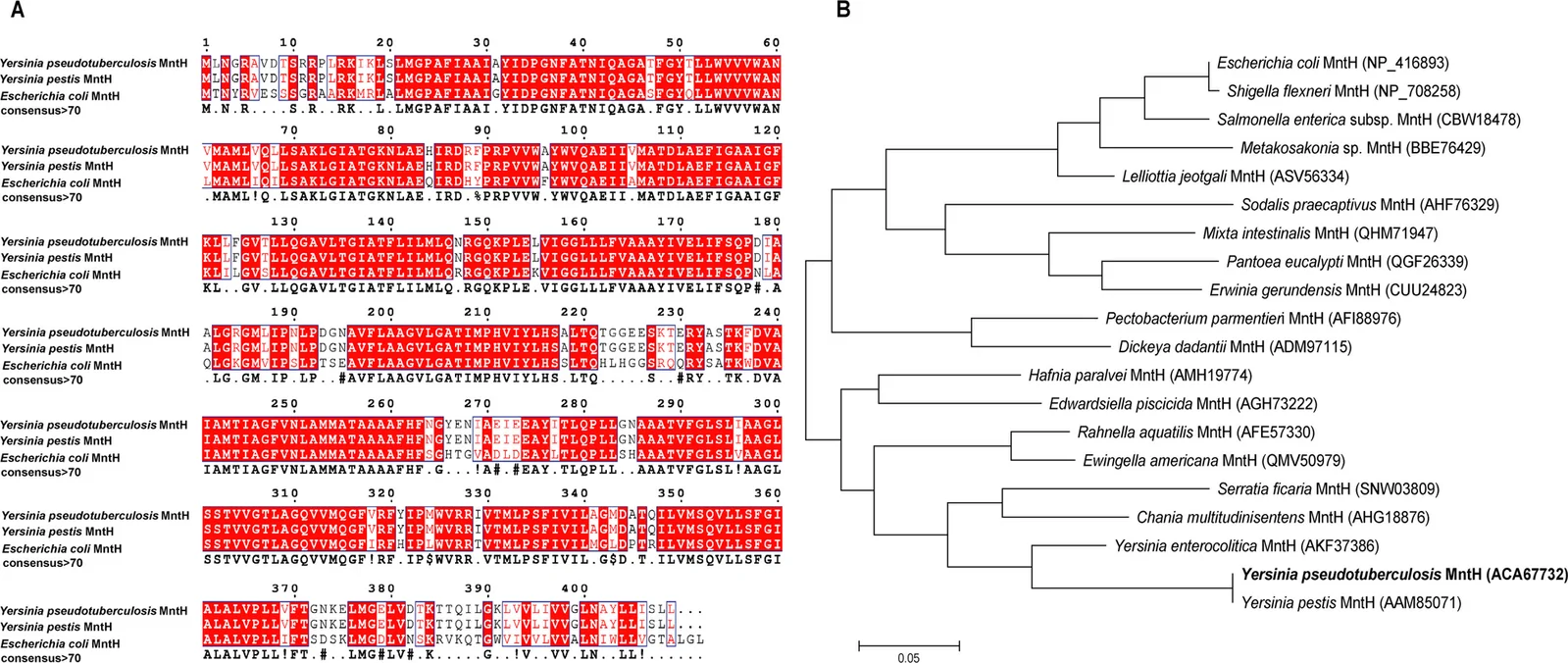
Bioinformatics analysis of MntH in *Yptb* YPIII strain. **A**. Comparison of MntH amino acid sequences. The amino acid sequences of the MntH homologs of *Yersinia pseudotuberculosis* YPIII (YPK_1439), *Yersinia pestis* (YP_2607) and *Escherichia coli* K-12 (B2392) were aligned using Clustal W and visualized using ESPript 3.0. **B**. Phylogenetic tree of MntH orthologs from different bacterial species. The phylogenetic tree was constructed by maximum likelihood with 1000 bootstrap replicates using MEGA 6.0 based on the alignment of MntH sequences. Scale lines indicate percent divergence (distance). The MntH of *Yptb* is shown in bold

MntH is a conserved Nramp-family manganese transporter reported in diverse bacteria, including *Escherichia coli*, *S.* Typhimurium, *Streptococcus mutans* and *Y. pestis* (Makui et al. 2000; Kehres et al. 2002, 2000; Kajfasz et al. 2020; Perry et al. 2012; Ha and Lee 2023). Genomic analyses of the *Yptb* YPIII strain identified an orthologous MntH transporter encoded by the gene *ypk_1439*, which shares 100.00% and 77.59% amino acid sequence identity with MntH in *Y. pestis* and *E. coli*, respectively (Fig. 1A). Further phylogenetic reconstruction revealed that *Yptb* MntH clustered tightly with orthologs from related bacterial species within a well-supported monophyletic clade, with the clustering pattern primarily reflecting taxonomic relatedness (Fig. 1B). Together with its high amino acid sequence identity to functionally characterized MntH proteins in *Y. pestis* and *E. coli*, this phylogenetic relationship suggests that *Yptb* MntH may play a conserved role in manganese homeostasis.

### Fur directly represses the expression of *mntH* in *Yptb*

**Fig. 2:**
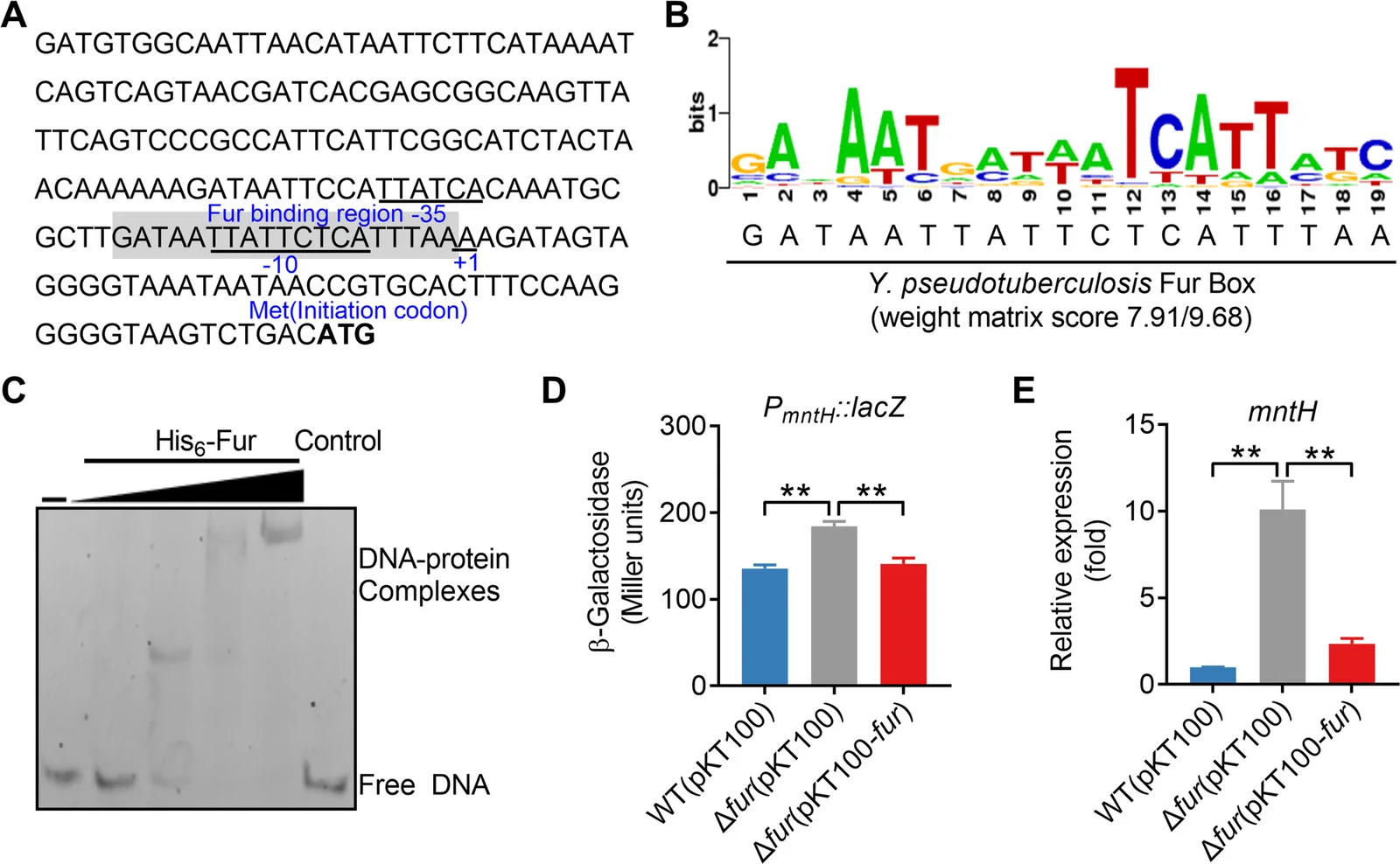
Fur negatively regulates *mntH* expression in *Yptb*. **A**. Identification of a Fur-binding site in the *mntH* promoter. The predicted Fur-binding site identified by Virtual Footprint is highlighted in gray. The putative − 35 and − 10 elements in the *mntH* promoter are underlined, and the ATG start codon is shown in bold. **B**. Virtual Footprint analysis of the Fur-binding sequence upstream of *mntH* in *Yptb* was conducted based on the consensus sequence for Fur binding from *E. coli*. The weight matrix score is 7.91. The plot displays information content (bits) on the Y-axis and nucleotide position on the X-axis. The Fur box is located at − 72 bp relative to the *mntH* gene. **C**. Fur binds to the promoter of *mntH*. EMSA was performed to examine the interaction between His_6_-Fur and the *mntH* promoter. Increasing concentrations of Fur (0, 0.2, 0.4, 0.6 and 0.8 µM) were incubated with 5 ng of the DNA probe. An equal amount of an unrelated 101-bp DNA fragment was used as a negative control. The EMSA was independently performed three times with consistent results, and one representative gel image is shown. **D**. Fur represses *mntH* expression. The *Yptb* WT, Δ*fur*, and Δ*fur*(*fur*) strains containing the* P*_*mntH*_*::lacZ* chromosomal transcriptional fusion reporter were grown to stationary phase in YLB medium, and the promoter activity of *mntH* was measured using β-galactosidase activity assays. **E**. The *Yptb* WT, Δ*fur*, and Δ*fur*(*fur*) strains were grown to exponential phase in YLB medium. RT-qPCR analysis of the relative expression of *mntH* was performed using 16S rRNA as an internal reference gene. Error bars represent ± SEM from three biological replicates, each comprising three technical replicates. Statistical significance was determined using one-way ANOVA followed by Dunnett’s multiple comparisons test. **P < 0.01

Metal acquisition systems are tightly regulated to balance nutritional needs and metal toxicity (Murdoch and Skaar 2022). In *Yptb*, which lacks homologs of the Mn^2+^-responsive regulators Mur and MntR, Fur governs iron and manganese homeostasis (Zuo et al. 2023). Analysis of the *mntH* promoter region identified a conserved 19-bp Fur binding motif (GATAATTATTCTCATTTAA) in its upstream region (Fig. 2A), displaying high similarity to the *E. coli* Fur box, with a weight matrix score of 7.91 (maximum score 9.68) (Fig. 2B). We further examined the interaction between Fur and the promoter of *mntH* using an electrophoretic mobility shift assay (EMSA). A DNA fragment of 101 bp upstream of *mntH*, designated *P*_*mntH*_, was amplified and used as the probe for Fur binding. As shown in Fig. 2C, incubation of the *P*_*mntH*_ probe with His_6_-Fur resulted in the formation of DNA–protein complexes.

To determine the role of Fur in regulating *mntH*, we integrated a *P*_*mntH*_*::lacZ* fusion reporter into the chromosome of *Yptb* wild-type (WT), Δ*fur* mutant, and Δ*fur*(*fur*) strain. β-galactosidase activity assays revealed that the *mntH* promoter activity was significantly elevated in the Δ*fur* mutant, and this phenotype was fully restored to WT levels in the Δ*fur*(*fur*) strain (Fig. 2D). We further investigated the role of Fur in regulating *mntH* expression using reverse-transcription quantitative PCR (RT-qPCR). The results showed that *mntH* expression was significantly increased in the Δ*fur* mutant, and this effect was fully reversed in the Δ*fur*(*fur*) strain (Fig. 2E). Collectively, these results establish Fur as a direct negative regulator of *mntH* by specifically binding to its promoter region.

### MntH is important for manganese and iron acquisition in *Yptb*

**Fig. 3:**
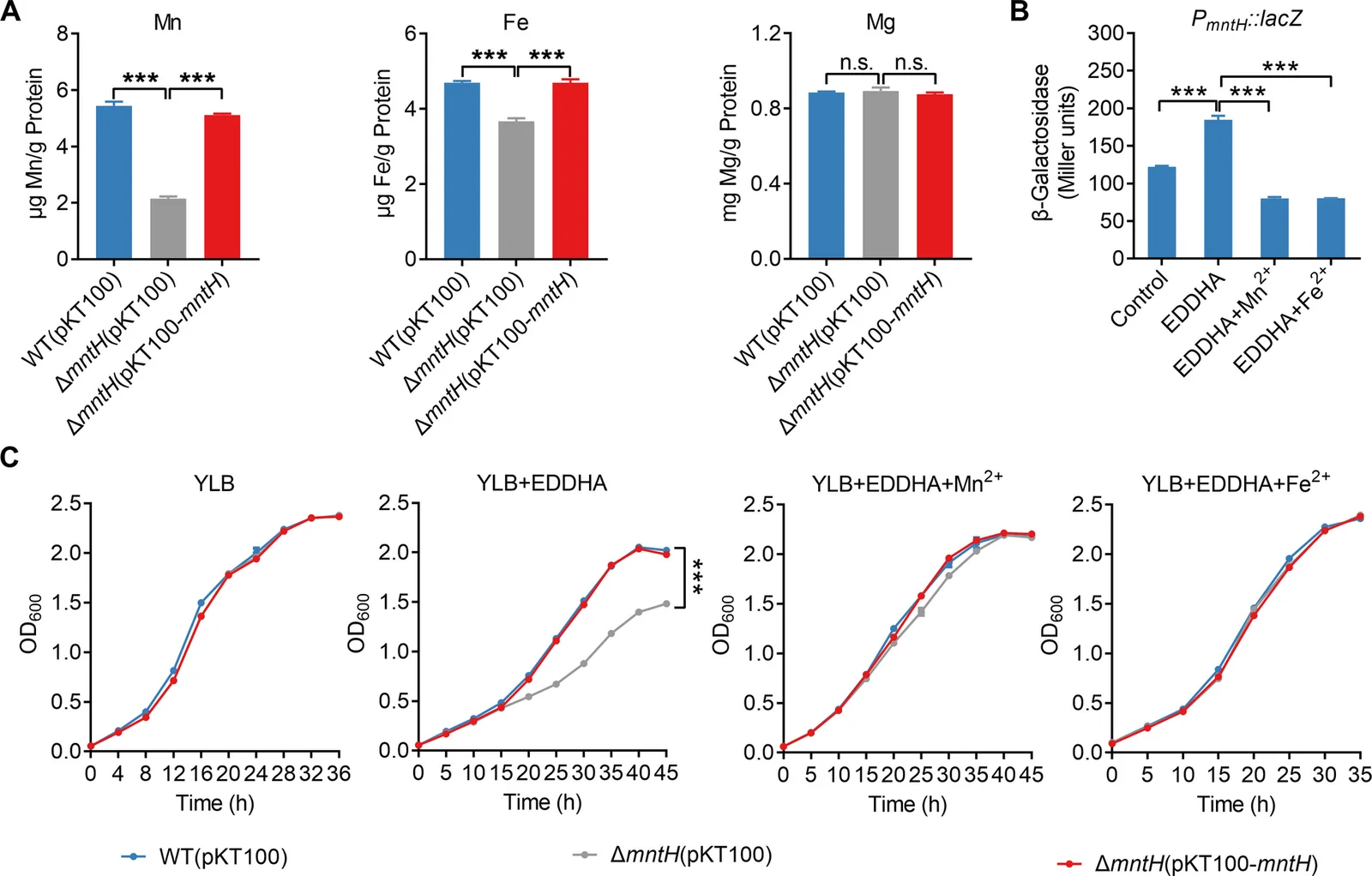
MntH is important for manganese and iron acquisition in *Yptb*. **A**. MntH is required for manganese and iron acquisition by *Yptb*. The *Yptb* WT, Δ*mntH*, and Δ*mntH*(*mntH*) strains were grown in M9G minimal medium to stationary phase, and the bacterial intracellular concentrations of manganese, iron, and magnesium were then measured using ICP-MS. **B**. *Yptb mntH* expression responds to Mn^2+^ and Fe^2+^. The *Yptb* WT strain containing the* P*_*mntH*_::*lacZ* chromosomal transcriptional fusion reporter was grown in YLB medium to exponential phase and then incubated for 2 h at 26°C in YLB medium containing no additives, 80 µM EDDHA alone, 80 µM EDDHA and 200 µM Mn^2+^, or 80 µM EDDHA and 200 µM Fe^2+^. The promoter activity of *mntH* was measured using β-galactosidase activity assays. **C**. MntH plays a critical role in supporting *Yptb* growth under ion-restricted conditions. Saturated bacterial cultures were diluted to an OD_600_ of 0.1 in YLB, YLB with 80 µM EDDHA, or YLB with 80 µM EDDHA and 200 µM Mn^2+^ or Fe^2+^. Growth was monitored by measuring the OD_600_ at the indicated time points. Error bars represent ± SEM from three biological replicates. Statistical significance was determined using one-way ANOVA followed by Dunnett’s multiple comparisons test. For the growth curves in Fig. 3 C, statistical analysis is presented for the final time point. ***P < 0.001; n.s., not significant

Given Fur's dual regulatory role and the established divalent cation transport function of MntH in *E. coli* and *S.* Typhimurium (Makui et al. 2000; Kehres et al. 2000), we investigated whether MntH in the *Yptb* YPIII strain facilitates both manganese and iron acquisition. Inductively coupled plasma mass spectrometry (ICP-MS) was used to quantify intracellular metal levels in *Yptb* WT, Δ*mntH* mutant, and Δ*mntH*(*mntH*) strain. The results showed that the Δ*mntH* mutant accumulated significantly lower levels of intracellular manganese and iron than the WT and Δ*mntH*(*mntH*) strains; in contrast, deletion of *mntH* had no effect on magnesium (Mg) accumulation (Fig. 3A).

To further determine whether the *mntH* expression responds to manganese and iron availability, we measured *P*_*mntH*_*::lacZ* reporter activity under different ion conditions. As shown in Fig. 3B, adding 80 µM of the metal chelator ethylenediamine-N,N′-bis(2-hydroxyphenylacetic acid) (EDDHA) to Yersinia-Luria–Bertani (YLB) medium significantly upregulated *mntH* expression. Importantly, this EDDHA-induced upregulation was markedly repressed by supplementing the medium with 200 µM Mn^2+^ or Fe^2+^. Given that Mn^2+^ and Fe^2+^ modulate *mntH* expression, we next investigated whether MntH is required for *Yptb* growth, particularly under ion-limiting conditions. Growth curves were determined for the *Yptb* WT, Δ*mntH* and Δ*mntH*(*mntH*) under conditions identical to those in Fig. 3B: YLB medium, YLB supplemented with 80 µM EDDHA, and EDDHA-containing YLB further supplemented with 200 µM Mn^2+^ or Fe^2+^. While Δ*mntH* exhibited no growth difference from the WT strain in YLB medium, its growth was significantly impaired upon the addition of 80 µM EDDHA to YLB (Fig. 3C). Notably, the growth defects of the Δ*mntH* mutant were rescued either by complementation with a plasmid expressing *mntH* or by supplementing 200 µM Mn^2+^ or Fe^2+^ (Fig. 3C). These results demonstrate that MntH plays a key role in supporting *Yptb* growth under ion-limited conditions.

### MntH contributes to *Yptb* survival under peroxide and hydroxyl radical-associated stress

**Fig. 4:**
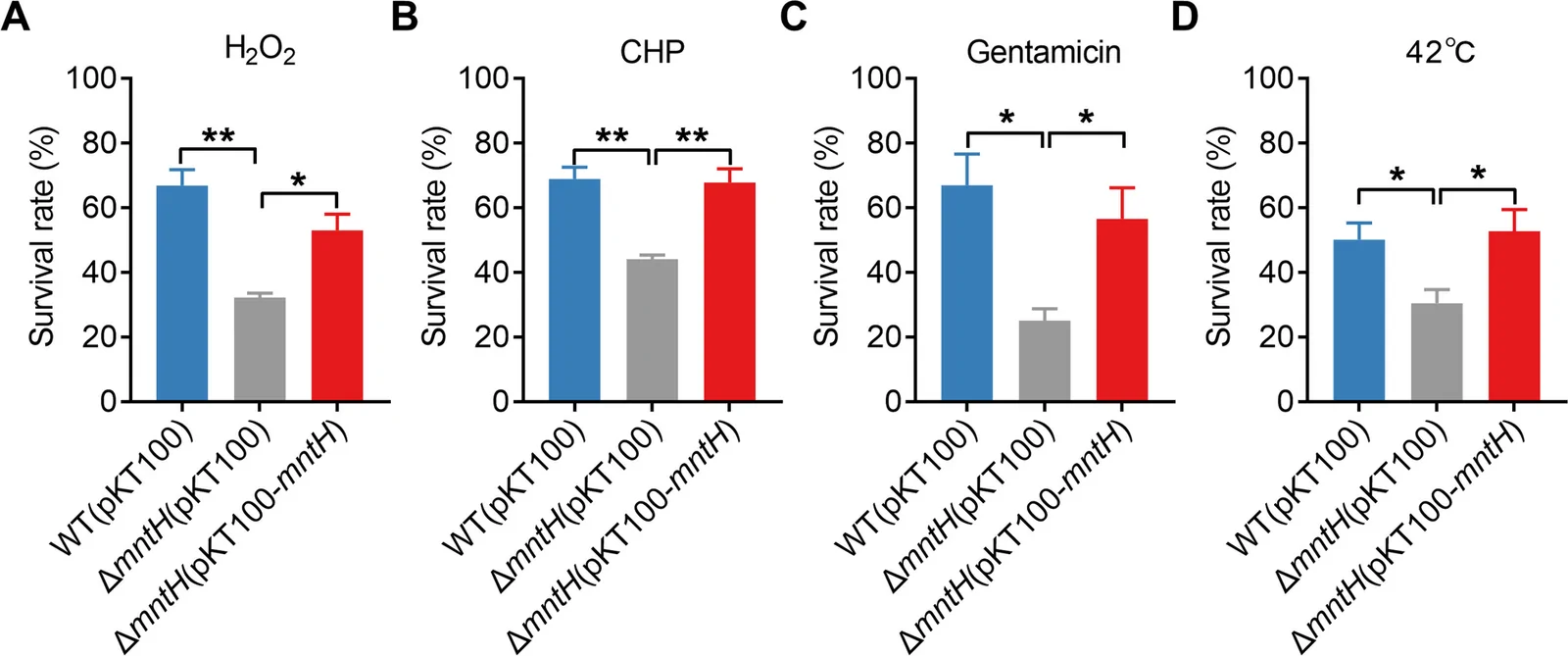
MntH promotes resistance to peroxide and hydroxyl radical-associated stress. **A-D**. The viability of relevant *Yptb* strains in mid-exponential phase was determined following exposure to 1 mM H_2_O_2_ (**A**), 0.5 mM CHP (**B**), 0.5 μg/mL gentamicin (**C**), and heat treatment at 42 °C (**D**) for 40 min. Error bars represent ± SEM from three independent biological replicates, each comprising three technical replicates. Statistical significance was determined using one-way ANOVA followed by Dunnett’s multiple comparisons test. **P < 0.01; *P < 0.05

MntH confers protection against hydrogen peroxide (H_2_O_2_)-induced oxidative stress in *E. coli* (Anjem et al. 2009). To determine whether MntH contributes to protecting *Yptb* from peroxide stress, we assessed the viability of *Yptb* WT, Δ*mntH* and Δ*mntH*(*mntH*) strains following exposure to H_2_O_2_ and cumene hydroperoxide (CHP). The results showed that the Δ*mntH* mutant was significantly more sensitive to H_2_O_2_ and CHP than the WT strain (Fig. 4A and B). Moreover, the survival rate of the complemented Δ*mntH*(*mntH*) strain was nearly restored to WT levels (Fig. 4A and B), indicating the important role of the MntH transport system in counteracting peroxide stress.

Gentamicin and high temperature are both known to induce the accumulation of hydroxyl radical (HR) (Kohanski et al. 2008; Matyushkina et al. 2026). To further explore MntH’s role in stress tolerance, we assessed the viability of the *mntH* mutant strain following challenge with 0.5 μg/mL gentamicin or heat treatment at 42 °C for 40 min. The Δ*mntH* mutant exhibited significantly greater sensitivity to gentamicin and high temperature than the WT strain (Fig. 4C and D). Notably, the survival rate of the complemented Δ*mntH*(*mntH*) strain was almost fully restored to WT levels (Fig. 4C and D). Together with the known ability of these stressors to induce HRs (Kohanski et al. 2008; Matyushkina et al. 2026), these results suggest that MntH contributes to *Yptb*’s ability to cope with HR-associated stresses induced by gentamicin and high temperature.

### MntH enhances the virulence of* Yptb* in infected mice

**Fig. 5:**
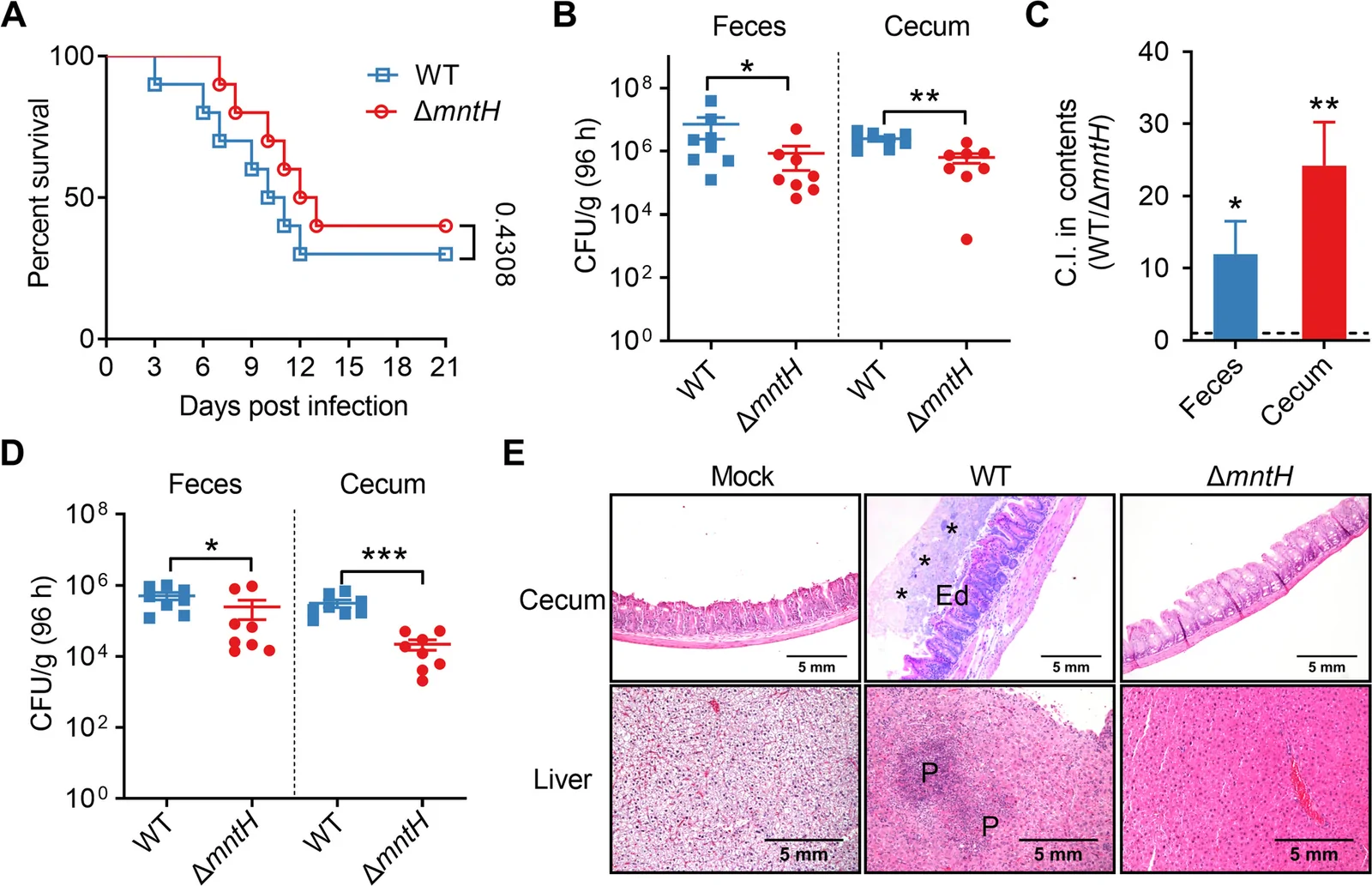
MntH contributes to the virulence of *Yptb* in a murine model. **A**-**E**. MntH contributes to *Yptb* virulence, colonization, and competitive fitness in mice. **A** Survival curves of female C57BL/6 mice (6–8 weeks old) orally infected with 1 × 10^9^ CFU of the indicated *Yptb* strains (*n* = 10 per group). **B** Bacterial burdens in the feces and cecum at 4 days post-infection were quantified by CFU assays (n = 8 per group). **C-D** For competitive infection assays, mice were co-infected with a 1:1 mixture of *Yptb* WT and Δ*mntH* strains (5 × 10^8^ CFU each). At 4 days post-infection, bacterial burdens in the feces and cecum were enumerated, and the competitive index (C.I.) was calculated as the output ratio (WT/Δ*mntH*) divided by the input ratio (WT/Δ*mntH*) (n = 8 per group). **E** H&E staining of cecal and liver tissues collected at 2 days post-infection. Representative images from 3–5 mice per group are shown. Asterisks (*): focal lymphocytic infiltration into the lamina propria; Ed: edema formation. P: piecemeal necrosis. The scale bar is 5 mm. Survival curves in Fig. 5 A were analyzed using the log-rank (Mantel-Cox) test. Data in Figs. 5 B-D are presented as the mean ± SEM. Statistical significance in Figs. 5 B and D was determined using the Mann–Whitney U test. Competitive indices in Fig. 5 C were analyzed using a two-tailed one-sample *t*-test against a theoretical value of 1. The dotted line indicates a competitive index of 1. *P < 0.05; **P < 0.01; ***P < 0.001

In light of the established role of MntH in the virulence of related pathogens, including *S.* Typhimurium, *Enterococcus faecalis* and* S. aureus* (Colomer-Winter et al. 2018; Ha and Lee 2023; Kehl-Fie et al. 2013), we assessed its contribution to *Yptb* pathogenesis. C57BL/6 mice were orogastrically infected with either the *Yptb* WT strain or Δ*mntH* mutant strain, and survival rates were analyzed. The results showed that the mortality rate in the Δ*mntH* mutant-infected group was lower than that in the WT-infected group, although the difference was not statistically significant (Fig. 5A). To further investigate the impact of MntH on *Yptb* colonization, we quantified bacterial loads in the cecum and feces of infected mice at 4 days post-infection. Consistently, mice infected with the Δ*mntH* mutant had significantly lower bacterial burdens in both sites relative to WT-infected mice (Fig. 5B).

To further assess the contribution of MntH to in vivo fitness, we performed a competitive infection assay between the WT and Δ*mntH* strains. Mice were co-infected with a 1:1 mixture of *Yptb* WT and Δ*mntH* mutant, and after 4 days, feces and cecum samples were collected for colony-forming unit (CFU) analysis. Following co-infection, the WT:Δ*mntH* ratios increased to ~ 12:1 in feces and ~ 24:1 in the cecum (Fig. 5C and D), suggesting that the *Yptb* WT exhibited greater competitive fitness than the Δ*mntH* mutant in the tissues of infected mice.

To characterize differences in infection outcomes between WT and Δ*mntH*, we examined histopathological changes in the cecum and liver of infected and control mice via hematoxylin and eosin (H&E) staining. WT infection induced more severe cecal inflammation than Δ*mntH* infection, with diffuse involvement of the lamina propria and cecal lymphoid follicles (Fig. 5E). Additionally, piecemeal necrosis was more pronounced in WT-infected mice compared with those infected with the Δ*mntH* mutant (Fig. 5E). These findings indicate that MntH contributes to more severe tissue pathology during *Yptb* infection. Collectively, these results support a role for MntH in *Yptb* colonization and infection-associated tissue damage.

### MntH helps *Yptb* evade CP-mediated growth inhibition

**Fig. 6:**
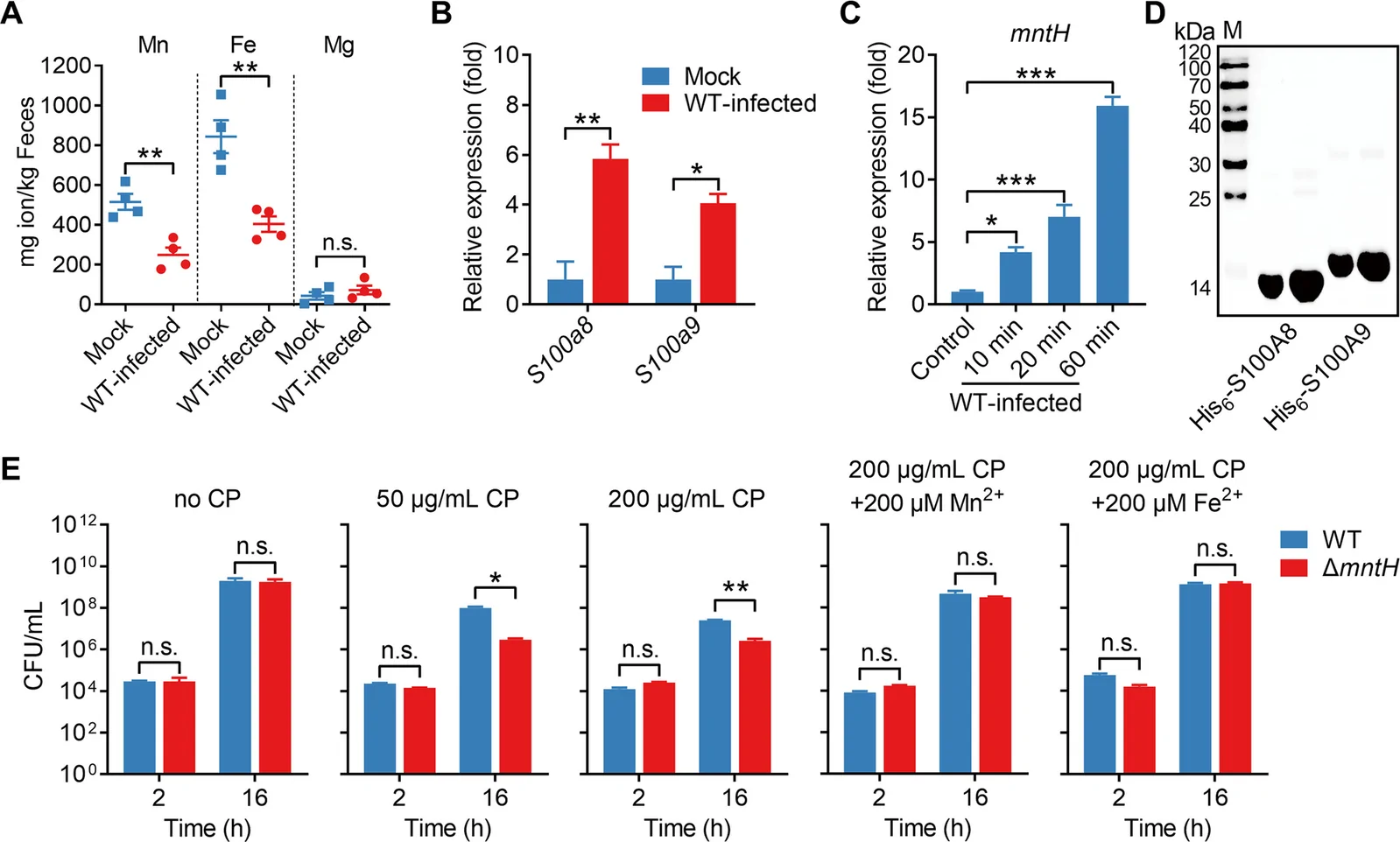
MntH is important for resistance of *Yptb* to calprotectin-mediated growth inhibition. **A**. Infection with *Yptb* WT strain decreases fecal manganese and iron levels in mice. Female C57BL/6 mice (6–8 weeks old) were orally infected with 1 × 10^9^ CFU of the *Yptb* WT strain. The fecal samples of infected mice were collected at 4 days post-infection, and ICP-MS was performed to measure the concentrations of manganese, iron, and magnesium. Four mice per group were used, and the data are presented as mean ± SEM. **B**. RT-qPCR analysis was performed to assess *S100a8* and *S100a9* expression in cecal tissues collected on day 4 from mice orally infected with 1 × 10^9^ CFU of *Yptb* WT or from uninfected control mice. *Gapdh* was used as the internal reference gene. Four mice per group were used, and the data are presented as mean ± SEM. **C**. The expression of the *mntH* gene was induced during infection. PMs from C57BL/6 mice were infected with *Yptb* WT at an MOI of 20 for the indicated time. RT-qPCR was performed to determine *mntH* expression in intracellular *Yptb* WT, using bacteria grown in YLB broth as the reference control. The 16S rRNA gene was used as an internal reference for normalization. **D**. Purification of S100A8 and S100A9 subunits of calprotectin. The S100A8 and S100A9 proteins, each with an N-terminal His_6_ tag, were purified using Ni^2+^-NTA affinity chromatography and subsequently subjected to 15% SDS-PAGE before being used in the growth inhibition assay. **E**. MntH helps *Yptb* evade calprotectin (CP)-induced growth inhibition. The *Yptb* strains were grown in YLB medium under the following conditions: no CP, 50 μg/mL CP, 200 μg/mL CP, 200 μg/mL CP with 200 μM Mn^2+^, or 200 μg/mL CP with 200 μM Fe^2+^. Bacterial growth was quantified by CFU enumeration. Quantitative data are presented as the mean ± SEM. Statistical significance in Figs. 6 A, B, and E was determined using the two-tailed unpaired Student’s *t*-test. Statistical significance in Fig. 6 C was determined using one-way ANOVA followed by Dunnett’s multiple comparisons test. ***P < 0.001; **P < 0.01; *P < 0.05; n.s., not significant

Given the important role of metal ion competition in bacterial evasion of host nutritional immunity (Hood and Skaar 2012), we assessed the impact of *Yptb* WT infection on fecal metal concentrations in mice using ICP-MS. The data revealed a significant depletion of fecal manganese and iron during infection: compared to uninfected controls, manganese levels decreased from 515 to 249 mg/kg and iron levels decreased from 843 to 404 mg/kg (Fig. 6A). Notably, magnesium levels remained unchanged, suggesting that *Yptb* employs a metal acquisition strategy specific to manganese and iron (Fig. 6A). We next examined the expression of CP subunit genes *S100a8* and *S100a9* in the cecum of *Yptb* WT-infected mice. Following RNA extraction, RT-qPCR analysis revealed significant upregulation of both *S100a8* and *S100a9* mRNA transcripts in *Yptb* WT-infected mice relative to uninfected controls (Fig. 6B). To evaluate bacterial adaptation to metal-restricted environments, we analyzed *mntH* expression in *Yptb* WT during infection. Peritoneal macrophages (PMs) from C57BL/6 mice were infected with *Yptb* WT at a multiplicity of infection (MOI) of 20, and *mntH* expression was quantified via RT-qPCR. Compared to *Yptb* WT grown in YLB broth (as a control), *mntH* was significantly upregulated in infected cells, with expression levels increasing over time post-infection (Fig. 6C).

Previous studies have shown that neutrophil-derived CP inhibits bacterial growth by chelating nutrient Mn^2+^ and other ions (Corbin et al. 2008; Kehl-Fie et al. 2011). To investigate whether *Yptb* can evade CP-mediated ion chelation via the MntH transporter, we prepared CP protein using the purified His-tagged S100A8 and S100A9 proteins (Fig. 6D), then monitored the growth of *Yptb* WT and Δ*mntH* mutant strains in nutrient-rich and CP-supplemented media. As shown in Fig. 6E, the Δ*mntH* mutant exhibited no growth difference compared to the WT strain in ion-rich YLB medium; however, its growth was significantly reduced in the presence of 50 or 200 µg/mL CP at later stages (16 h). Notably, the growth defects of the Δ*mntH* mutant were alleviated by supplementing YLB medium with 200 µM Mn^2+^ or Fe^2+^ alongside 200 µg/mL CP, indicating that MntH-mediated metal transport mitigates CP-induced ion starvation. Collectively, these findings suggest that the MntH transporter enables *Yptb* to overcome CP-mediated growth inhibition by effectively acquiring manganese and iron.

## Discussion

Bacterial pathogens depend on metal ions to replicate and survive within the host, which in turn employs many nutritional immunity strategies to restrict their access to these essential nutrients (Hood and Skaar 2012). However, the mechanisms by which pathogens navigate and evade these host defenses remain incompletely defined. In this study, we identified MntH, a conserved Nramp-family transporter in the *Yptb* YPIII strain, whose expression is directly repressed by Fur. We demonstrate that MntH facilitates dual acquisition of manganese and iron, enhances resistance to environmental stresses, and promotes in vivo virulence and fitness. Notably, MntH facilitates the evasion of CP-mediated growth inhibition by restoring metal import, even in the face of host CP upregulation induced by infection. These findings deepen our understanding of the adaptive strategies by which *Yptb* navigates host-imposed metal limitation during infection.

While MntH is traditionally recognized as a manganese transporter across various bacterial species (Bosma et al. 2021), such as *Bradyrhizobium japonicum* (Hohle et al. 2011; Hohle and O'Brian 2009), *Listeria monocytogenes* (Wu et al. 2023), *Y. pestis* (Perry et al. 2015) and *Brucella abortus* (Anderson et al. 2009), emerging evidence indicates that MntH in *E. coli* and *S.* Typhimurium can transport both Mn^2+^ and Fe^2+^, facilitating adaptation to fluctuating metal availability (Makui et al. 2000; Kehres et al. 2000). Our results show that *Yptb* MntH also mediates the acquisition of manganese and iron, thereby supporting bacterial growth under metal-restricted conditions (Fig. 3). Due to the potential toxicity of Mn at elevated concentrations, precise regulation of MntH expression is required (Waters 2020). Some manganese-responsive regulators, such as Mur, can regulate the expression of MntH in *B. abortus* (Anderson et al. 2009), while MntR is involved in regulating MntH expression in *E. coli* (Waters et al. 2011), *B. subtilis* (Que and Helmann 2000), and *S.* Typhimurium (Ikeda et al. 2005). In addition, MntH is also regulated by Fur in *E. coli*, *S.* Typhimurium (Kehres et al. 2000), *Y. pestis* (Perry et al. 2012), and *Shigella flexneri* (Runyen-Janecky et al. 2006). Interestingly, our previous studies have shown that Fur functions as a dual sensor for both Mn^2+^ and Fe^2+^ in *Yptb*, which lacks homologs of Mur and MntR (Zuo et al. 2023). Here, we further show that Fur negatively regulates *mntH* expression and links *mntH* transcription to extracellular manganese and iron availability (Fig. 3B).

Manganese helps bacteria withstand oxidative stress by serving as a cofactor for reactive oxygen species-detoxifying enzymes, including superoxide dismutase and catalase (Aguirre et al. 2013; Wan et al. 2017; Diaz-Ochoa et al. 2016). Our observation that the Δ*mntH* mutant was more sensitive to H_2_O_2_ and CHP indicates that MntH contributes to peroxide stress resistance (Fig. 4A and B). In addition, the increased sensitivity of Δ*mntH* to gentamicin and high temperature (Fig. 4C and D), two stressors associated with HR accumulation (Kohanski et al. 2008; Matyushkina et al. 2026), suggests that MntH may also support tolerance to HR-associated stresses. Moreover, metal ion transport systems are important for pathogen adaptation and virulence within the host environment (Porcheron et al. 2013). We next examined whether this specific transport system also contributes to the virulence and in vivo fitness of *Yptb*. The results showed that the mortality rate in the Δ*mntH* mutant-infected group was lower than that in the WT-infected group, although the difference was not statistically significant (Fig. 5A), while bacterial loads in the cecum and feces were significantly reduced at 4 days post-infection (Fig. 5B). In competition assays, WT outgrew Δ*mntH* in feces and cecum (Fig. 5C and D), indicating a significant fitness advantage. Histological analysis further revealed that the Δ*mntH* mutant induced only mild cecal inflammation and less hepatic necrosis than the WT strain (Fig. 5E). These results collectively indicate that MntH contributes to colonization and infection-associated tissue damage during *Yptb* infection.

CP-mediated nutritional immunity imposes strong metal limitation during infection, and several pathogens counter this pressure through dedicated metal acquisition systems (Diaz-Ochoa et al. 2016; Kehl-Fie et al. 2013; Hood and Skaar 2012). In *Yptb* YPIII, previously identified manganese-, zinc-, and iron-acquisition systems contribute to immune evasion (Zhu et al. 2021; Liu et al. 202﻿3; Dai et al. 2026), but the role of MntH in the *Yptb* YPIII strain under CP-imposed metal restriction remained unclear. Our data show that the *Yptb* YPIII strain triggers an inflammatory response that upregulates CP expression and reduces fecal levels of iron and manganese, but not magnesium (Fig. 6A and B). Reduced availability of metals in the gut has also been reported during infections with *S*. Typhimurium and *Clostridioides difficile* (Diaz-Ochoa et al. 2016; Lopez et al. 2019), suggesting that metal restriction in the gut is a common host defense strategy. Notably, *mntH* expression increased over time during infection of peritoneal macrophages (Fig. 6C), suggesting a response to metal limitation. Functional assays further demonstrated that, under 200 µg/mL CP treatment, the Δ*mntH* mutant exhibited a significant growth defect relative to the WT at 16 h, and this defect was alleviated by Mn or Fe supplementation (Fig. 6E). These findings indicate that MntH facilitates metal acquisition under CP-mediated stress and plays a key role in sustaining bacterial growth during host-imposed nutritional immunity.

## Conclusion

In this study, we demonstrate that MntH is important for the uptake of manganese and iron in *Yptb* under host-imposed metal limitation. Fur directly represses *mntH* expression, orchestrating a precise regulatory balance between metal acquisition and cellular demand. In addition to its pivotal role in metal transport, MntH enables *Yptb* to survive peroxide and HR-associated stresses and overcome CP-mediated nutritional immunity, thereby promoting colonization and tissue damage in vivo. Collectively, these findings identify Fur-regulated MntH as an important contributor to metal homeostasis, stress tolerance, and gut fitness in *Yptb*. Future studies should define how MntH coordinates with other metal acquisition systems under host-imposed nutritional immunity.

## Materials and methods

### Bacterial strains and growth conditions

Bacterial strains and plasmids used in this study are listed in Supplementary Table 1. *E. coli* strains were cultured in Luria–Bertani (LB) medium at 37 °C. *Yptb* strains were grown in YLB broth (1% tryptone, 0.5% yeast extract, 0.5% NaCl) or M9G minimal medium (Na_2_HPO_4_, 6 g L^−1^; KH_2_PO_4_, 3 g L^−1^; NaCl, 0.5 g L^−1^; NH_4_Cl, 1 g L^−1^; MgSO_4_, 2 mM; CaCl_2_, 0.1 mM; 0.2% glucose, pH 7.0) at 26°C, as previously described (Li et al. 2021). *Yptb* YPIII was the parental strain for all derivatives used in this study. In-frame deletions were generated as described previously (Zhu et al. 2021). Bacterial growth was assessed by measuring the optical density at 600 nm (OD_600_). Antibiotics were used at the following concentrations: nalidixic acid, 20 µg/mL; kanamycin, 50 µg/mL; and chloramphenicol, 20 µg/mL.

### Plasmid construction

The primers used in this study are listed in Supplementary Table 2. The Δ*mntH* in-frame deletion mutant was constructed using the suicide vector pDM4 as previously described (Liu et al. 2023). For complementation, the *mntH* coding sequence was amplified from *Yptb* genomic DNA, cloned into pKT100, and introduced into the Δ*mntH* mutant by electroporation. To generate pDM4-*PmntH*::*lacZ*, the 459-bp *mntH* promoter region was amplified from *Yptb* genomic DNA and used to replace the *T6SS-4p* promoter region in the previously constructed pDM4-*T6SS-4p*::*lacZ* reporter plasmid (Zhu et al. 2026). The pET28a-*S100a8* and pET28a-*S100a9* expression plasmids were generated by cloning synthesized *S100a8* and *S100a9* fragments into pET28a. All constructs were confirmed by DNA sequencing.

### Sequence alignment and phylogenetic analysis

The sequence alignment and phylogenetic analysis were conducted as described (Liu et al. 2025). Briefly, the amino acid sequence of *Yptb* MntH was used as the query for an NCBI BLASTP search. To reduce sequence redundancy, highly similar sequences from closely related strains were excluded, and representative MntH homologs spanning distinct genera and families within the order *Enterobacterales* were selected for phylogenetic analysis. Amino acid sequence alignment of MntH was generated using ClustalW in MEGA 6.0 software with default parameters. The alignment was manually trimmed and subsequently visualized using ESPript 3.0. The phylogenetic tree was constructed based on the aligned sequences using the maximum-likelihood algorithm in MEGA 6.0, with 1,000 bootstrap replicates.

### Overexpression and purification of recombinant proteins

Overexpression and purification of recombinant proteins were performed as previously described (Liu et al. 2025) with minor modifications. Plasmids pET28a-*fur*, pET28a-*S100a8*, and pET28a-*S100a9* were transformed into BL21 (DE3). Cultures were grown in LB medium to an OD_600_ of 0.5 and induced with 0.3 mM isopropyl‑β‑D‑thiogalactopyranoside (IPTG) for approximately 12 h at 24 °C for His_6_-Fur or 16 °C for His_6_-S100A8 and His_6_-S100A9. Cells were disrupted by sonication, and recombinant proteins were purified using His-Bind nickel-nitrilotriacetic acid resin (Novagen, Madison, WI, United States). Eluted His_6_-Fur was dialyzed against buffer (50 mM Tris, 137 mM NaCl, 10% glycerol, pH 7.5), whereas His_6_-S100A8 and His_6_-S100A9 were dialyzed against 1× PBS containing 10% glycerol (pH 7.5) at 4 °C. The resulting proteins were stored at − 80 °C until use. Protein concentrations were determined via the Bradford assay (Bio-Rad, Hercules, CA, United States) with bovine serum albumin as the standard.

### Electrophoretic mobility shift assay (EMSA)

EMSA was performed as previously described (Li et al. 2021). The *P*_*mntH*_ fragment was amplified from the *Yptb* YPIII genome via PCR using the primer pair *P*_*mntH*_-F/*P*_*mntH*_-R. Purified His_6_-Fur (0, 0.2, 0.4, 0.6 and 0.8 µM) was incubated with 5 ng of the DNA probe in EMSA buffer (20 mM Tris–HCl, pH 7.4, 4 mM MgCl_2_, 100 mM NaCl, 100 µM MnCl_2_, 1 mM dithiothreitol, 10% glycerol). Following a 20-min incubation at room temperature, the binding reaction mixture was subjected to electrophoresis on a 6% native polyacrylamide gel with 5% glycerol in 0.5 × Tris–borate-EDTA buffer. DNA probes were visualized using SYBR Safe DNA gel stain (Invitrogen, Grand Island, NY, United States). A 101-bp unrelated DNA fragment was included as a negative control in the binding reactions.

### β-Galactosidase assay

The *lacZ* fusion reporter vector pDM4-*P*_*mntH*_*::lacZ* was transformed into *E. coli* S17-1 *λpir* and subsequently mated with relevant *Yptb* strains, as previously described (Li et al. 2021). Reporter strains were cultured in YLB medium at 26°C under the specified conditions until they reached either exponential or stationary phase. β-galactosidase activity was measured using o-nitrophenyl β-D-galactopyranoside (ONPG) as the substrate.

### Reverse-transcription quantitative PCR (RT-qPCR)

Reverse-transcription quantitative PCR (RT-qPCR) was performed as previously described (Zhu et al. 2021). Total RNA was extracted from exponential-phase bacterial cultures and from cecal tissues of infected or uninfected mice (n = 4/group; 4 days post-infection) with on-column DNase I digestion. First-strand cDNA was synthesized from 1 μg of total RNA. RT-qPCR was performed using primers listed in Supplementary Table 2 to quantify cecal *S100a8 and S100a9* expression normalized to *Gapdh* and bacterial *mntH* expression normalized to 16S rRNA. All samples were analyzed in triplicate, and relative expression was calculated using the 2^−ΔΔCt^ method.

### Measurement of metal concentrations by ICP-MS

Bacterial intracellular ion levels were determined as previously described (Li et al. 2022). Briefly, 20 mL of stationary-phase *Yptb* cultures grown in M9G medium were collected and washed twice with M9G, and the collected pellets were lysed with BugBuster (Novagen, Madison, WI, USA) with rotation at 4 °C overnight. Total protein for each sample was measured using the NanoDrop ND-1000 spectrophotometer (NanoDrop Technologies) and then diluted 10-fold in 2% molecular grade nitric acid to a total volume of 5 mL. Samples were analyzed by ICP-MS and corrected for appropriate buffers and dilution factors. To determine the effects of *Yptb* WT infection on manganese, iron, and magnesium levels in mice, six-to-eight-week-old female C57BL/6 mice were intragastrically inoculated with 1 × 10^9^ CFU of the indicated *Yptb* WT strain. Fecal samples collected 4 days post-infection were analyzed by ICP-MS to quantify manganese, iron, and magnesium concentrations (n = 4/group).

### Bacterial survival assays

Bacterial survival assays were performed as previously described by Li (Li et al. 2021), with minor modifications. *Yptb* strains were grown in YLB medium to mid-exponential phase, then collected, washed, and diluted 50-fold in M9G medium. Cells were treated with 1 mM H_2_O_2_, 0.5 mM CHP, or 0.5 μg/mL gentamicin at 26°C for 40 min, or subjected to heat treatment at 42 °C for 40 min. After treatment, cultures were serially diluted, plated onto YLB agar plates, and incubated at 26°C for 36 h before colony enumeration. Survival percentage was calculated as (CFU from stressed cells / CFU from unstressed cells) × 100%.

### Murine infection

Murine infection experiments were performed as previously described by Liu (Liu et al. 2025). All animal procedures were approved by the Animal Welfare and Research Ethics Committee of Northwest A&F University (Protocol number: XN2023-1004). 6–8-week-old female C57BL/6 mice were purchased from SPF Biotechnology Co., Ltd. (Beijing, China) and acclimatized for 3–5 days before infection. *Yptb* strains were cultured in YLB medium at 26°C to mid-exponential phase, washed twice with sterilized phosphate-buffered saline (PBS), and used to orally gavage mice at a dose of 1 × 10^9^ CFU per mouse. At 4 days post-infection, mice were euthanized and the CFUs of each *Yptb* strain in collected feces and cecum were enumerated on selective YLB agar plates containing antibiotics. For survival assays, 1 × 10^9^ CFU of each strain was administered to separate mouse groups, and their survival was monitored daily for 21 days. For competitive infection assays, mice were orally co-infected with a 1:1 mixture of the *Yptb* WT strain and Δ*mntH* strain (5 × 10^8^ CFU each), and the competitive index (C.I.) was calculated from fecal and cecal bacterial loads at 4 days post-infection as the output ratio divided by the input ratio (n = 8).

### Isolation, culture and infection of peritoneal macrophages (PMs) and RT-qPCR

PMs were isolated from mice following a 3-day intraperitoneal elicitation with beef extract peptone medium (0.3% beef extract, 1% peptone, 0.5% NaCl). Harvested PMs were cultured for 1 day in RPMI 1640 medium containing 10% fetal bovine serum (FBS), penicillin (100 U/mL), streptomycin (100 μg/mL), sodium pyruvate (10 μM), L-Glutamine (2 mM), non-essential amino acids (0.1 mM), 2-mercaptoethanol (50 μM), and HEPES (25 mM). For infection, cultured PMs were exposed to *Yptb* strains at an MOI of 20 (Zhu et al. 2021). At designated time points, infected macrophages were rinsed three times with Hank’s Balanced Salt Solution to eliminate extracellular bacteria. Total RNA was subsequently isolated using the RNA-queous Total RNA Isolation Kit (Invitrogen) following the manufacturer’s protocol. Quantification of specific mRNA transcripts was performed using SYBR Green Real-Time PCR Master Mix (Life Technologies), with YLB-grown bacterial cultures used as reference controls. Expression levels were normalized to 16S rRNA to ensure data consistency.

### Histology analysis

Histological analysis was performed as previously described (Zuo et al. 2023). *Yptb* strains were grown to mid-exponential phase in YLB medium at 26°C, washed twice with sterile PBS, and resuspended. Mice were infected orogastrically with 1 × 10^9^ CFU of the respective strain using a ball-tipped feeding needle; control mice received PBS. Two days post-infection, cecal and liver tissues were collected from PBS control and *Yptb*-infected mice. Tissues were fixed in 4% formalin for 24–48 h, paraffin-embedded, sectioned at 3 µm, and stained with hematoxylin and eosin (H&E). Tissue sections from 3–5 mice per group were analyzed in a blinded manner by light microscopy.

### Bacterial growth assay

The CP-mediated growth assay was performed as previously described (Diaz-Ochoa et al. 2016) with minor modifications. The purified proteins S100A8 and S100A9 were resuspended in Hanks-HEPES buffer containing 1.3 mM calcium. Subsequently, equimolar amounts of S100A8 and S100A9 proteins were mixed and incubated at room temperature for 30 min to allow the formation of the CP complex, which was then added to the culture medium. Both *Yptb* WT and Δ*mntH* strains were grown to late exponential phase. Then, these cultures were inoculated at 1% (v/v) into fresh YLB medium under the following conditions: no CP, 50 μg/mL CP, 200 μg/mL CP, 200 μg/mL CP with 200 μM Mn^2+^, or 200 μg/mL CP with 200 μM Fe^2+^. CFU were enumerated by plating serial dilutions on YLB agar plates containing nalidixic acid after 2 and 16 h of growth. For growth assays under ion-restricted conditions, saturated bacterial cultures were diluted to an OD_600_ of 0.1 in YLB, YLB with 80 µM EDDHA, or YLB with 80 µM EDDHA and 200 µM Mn^2+^ or Fe^2+^, and growth was monitored by measuring the OD_600_ at the indicated time points.

### Statistical analysis

All statistical analyses were performed using GraphPad Prism 8.0.2 (GraphPad Software, San Diego, CA, USA). Survival curves were analyzed using the log-rank (Mantel-Cox) test, and growth curve comparisons were performed at the final time point. Competitive indices were analyzed using a two-tailed one-sample *t*-test against a theoretical value of 1. Other comparisons were performed using the two-tailed unpaired Student’s *t*-test, Mann–Whitney U test, or one-way ANOVA followed by Dunnett’s multiple comparisons test, as specified in the figure legends. Data are presented as mean ± standard error of the mean (SEM), unless otherwise indicated. Statistical significance was defined as follows: *** *P* < 0.001; ** *P* < 0.01; * *P* < 0.05; n.s., not significant.

## Supplementary Information

Supplementary Material 1.

## Data Availability

The data that support the findings of this study are available in the figures, tables, and supplementary material of this article.

## Abbreviations

ANOVA: Analysis of variance
CFU: Colony-forming unit
C.I.: Competitive index
CP: Calprotectin
CHP: Cumene hydroperoxide
EDDHA: Ethylenediamine-N,N′-bis(2-hydroxyphenylacetic acid)
EMSA: Electrophoretic mobility shift assay
Fe: Iron
Fur: Ferric uptake regulator
H&E: Hematoxylin and eosin
H_2_O_2_: Hydrogen peroxide
HR: Hydroxyl radical
ICP-MS: Inductively coupled plasma mass spectrometry
IPTG: Isopropyl‑β‑D‑thiogalactopyranoside
MOI: Multiplicity of infection
Mn: Manganese
Nramp: Natural resistance-associated macrophage protein
ONPG: O-nitrophenyl β-D-galactopyranoside
PBS: Phosphate-buffered saline
PMs: Peritoneal macrophages
RT-qPCR: Reverse-transcription quantitative PCR
SEM: Standard error of the mean
T6SS: Type VI secretion system
WT: Wild type
*Yptb*: *Yersinia pseudotuberculosis*
YLB: Yersinia-Luria–Bertani
