# Supplementary material for: Fur-regulated MntH mediates metal homeostasis and immune evasion in Yersinia pseudotuberculosis
Source: Stress Biol. 2026 Nov 11;6(1):73. doi: 10.1007/s44154-026-00348-8 (PMC13755157; doi:10.1007/s44154-026-00348-8)
Supplement: Supplementary file 1 [file 44154_2026_348_MOESM1_ESM.docx]

**Supplementary Information**

**Fur-regulated MntH mediates metal homeostasis and immune evasion in *Yersinia pseudotuberculosis***

Peishuai Fu^#^, Fengge Song^#^, Nuoping Xu, Jitong Jiao, Liya Zhang, Xiaofang Ma, Danyang Yu, Yuqi Liu, Yuxin Zuo, Yantao Yang, Zhiyan Wei, Yao Wang, Xihui Shen, Changfu Li*, Lingfang Zhu*

Shaanxi Key Laboratory of Agricultural and Environmental Microbiology, College of Life Sciences, Northwest A&F University, Yangling, Shaanxi 712100, P. R. China

^#^These authors contributed equally to this work.

***For correspondence:**

Changfu Li (lierfu@nwafu.edu.cn);

Lingfang Zhu (lingfangzhu@nwafu.edu.cn).

**Running title:** Fur-regulated MntH enhances bacterial adaptability.

**This PDF file includes:**

Supplementary Tables 1-2;

Supplementary References.

**Supplementary Tables**

**Table 1 Bacterial strains and plasmids**

| **Strain or plasmid** | **Relevant characteristics** | **Reference** |
| --- | --- | --- |
| ***E. coli*** | | |
| S17-1*λ pir* | *λ-pir* lysogen of S17-1, *thi pro hsdR hsdM^+^ recA* RP4 -Tc::Mu-Km::Tn7 | (Simon et al. 1983) |
| BL21(DE3) | Host for expression vector pET28a | Novagen |
| DH5α | FΦ80Δ*lacZ*ΔM15/Δ(*lacZYA-argF*)*U169recA1 endA1 hsdR17* | Novagen |
| ***Y. pseudotuberculosis*** | | |
| WT | Wild-type *Y. pseudotuberculosis* pIB1(*Yptb*), Nal^r^ | (Rosqvist et al. 1988) |
| Δ*mntH* | *mntH* (*ypk_1439*) gene deleted in *Yptb*, Nal^r^ | This study |
| Δ*fur* | *fur* (*ypk_2991*) gene deleted in *Yptb*, Nal^r^ | (Zuo et al. 2023) |
| WT(Vector) | WT(Vector) containing pKT100, Nal^r^, Km^r^ | This study |
| Δ*mntH*(Vector) | Δ*mntH* containing pKT100, Nal^r^, Km^r^ | This study |
| Δ*mntH*(*mntH*) | Δ*mntH* containing pKT100-*mntH*, Nal^r^, Km^r^ | This study |
| Δ*fur*(Vector) | Δ*fur* containing pKT100, Nal^r^, Km^r^ | (Zuo et al. 2023) |
| Δ*fur*(*fur*) | Δ*fur* containing pKT100-*fur*, Nal^r^, Km^r^ | (Zuo et al. 2023) |
| **Plasmid** | | |
| pKT100 | Cloning vector, p15A replicon, Km^r^ | (Hu et al. 2009) |
| pKT100-*mntH* | *mntH* under the control of chloramphenicol resistance gene promoter in plasmid pKT100, Km^r^ | This study |
| pKT100-*fur* | *fur* under the control of chloramphenicol resistance gene promoter in plasmid pKT100, Km^r^ | (Zuo et al. 2023) |
| pET28a | Expression vector with N-terminal hexahistidine affinity tag, Km^r^ | Novagen |
| pET28a-*fur* | pET28a carrying *fur* coding region, Km^r^ | (Zuo et al. 2023) |
| pET28a-*S100a8* | pET28a carrying *S100A8* coding region, Km^r^ | This study |
| pET28a-*S100a9* | pET28a carrying *S100A9* coding region, Km^r^ | This study |
| pDM4 | Suicide vector, *mob*RK2, *ori*R6K, *pir*, *sacB*, Cm^r^ | (Milton et al. 1996) |
| pDM4-Δ*mntH* | Construct used for in-frame deletion of *mntH*, Cm^r^ | This study |
| pDM4-*T6SS-4p*::lacZ | For *T6SS-4* promoter fusion to *Yptb*, Cm^r^ | (Zhu et al. 2026) |
| pDM4-*P_mntH_*::*lacZ* | For *mntH* promoter fusion to *Yptb*, Cm^r^ | This study |

*Nal^r^, Km^r^ and Cm^r^ represent resistance to Nalidixic acid, Kanamycin and Chloramphenicol at 20, 50 and 20 μg ml^-1^, respectively.

**Table 2 Primers used in this study.**

| **Primers** | **5’-3’ sequence** | **Function** |
| --- | --- | --- |
| *mntH-*1F-BglII | GAAGATCTCATTGCTGTTGCCGCCATG | To generate  pDM4-Δ*mntH* |
| *mntH-*1R | GGGCGACGAGATGTATCGACA |  |
| *mntH-*2F | **TGTCGATACATCTCGTCGCCC**TGGTGGTTCTGATCGTGGTTG |  |
| *mntH-*2R-SalI | GCGTCGACCCGTTGGCTGGCGGTTTAT |  |
| *mntH-*F*-*SphI | ACATGCATGCATGCTTAATGGCCGTGCTGT | To generate  pKT100-*mntH* |
| *mntH-*R*-*SalI | GCGTCGACCTAAAGCAGGCTAATCAGTAAATAG |  |
| *P_mntH_-*F-SalI | GCGTCGACGGACATATGTGCTCCAAA | To generate  pDM4-*P_mntH_*::*lacZ* |
| *P_mntH_-*R-XbaI | TGCTCTAGACATGCCATTAAGCATGTCAGA |  |
| *P_mntH_*-F | ATAATTCCATTATCACAAATGCG | For EMSA |
| *P_mntH_*-R | CATGTCAGACTTACCCCCTTGGA |  |
| control-F | TATTCGGTCATCCTGCTTG |  |
| control-R | CCTTCGGCATCTTCCATC |  |
| Q16S-F | CTAGCGATTCCGACTTCAT | For RT-qPCR |
| Q16S-R | CCCTTATCCTTTGTTGCC |  |
| Q*mntH*-F | CTGCTGTCCGCCAAGCT |  |
| Q*mntH*-R | AATTTCGGCTTGCACCC |  |
| *Gapdh*-F | CATCACTGCCACCCAGAAGACTG |  |
| *Gapdh*-R | ATGCCAGTGAGCTTCCCGTTCAG |  |
| Q*S100a8*-F | TGTCCTCAGTTTGTGCAGAATATAAA |  |
| Q*S100a8*-R | TCACCATCGCAAGGAACTCC |  |
| Q*S100a9*-F | GGTGGAAGCACAGTTGGCA |  |
| Q*S100a9*-R | GTGTCCAGGTCCTCCATGATG |  |

Underlined sites indicate restriction enzyme cutting sites added for cloning. Letters in boldface denote the annealing regions for overlap PCR.

**Supplementary References**

Hu Y, Lu P, Wang Y, Ding L, Atkinson S, Chen S (2009) OmpR positively regulates urease expression to enhance acid survival of *Yersinia pseudotuberculosis*. Microbiology (Reading) 155(Pt 8):2522-2531. <https://doi.org/10.1099/mic.0.028381-0>

Milton DL, O'Toole R, Horstedt P, Wolf-Watz H (1996) Flagellin A is essential for the virulence of *Vibrio anguillarum*. J Bacteriol 178(5):1310-1319. <https://doi.org/10.1128/jb.178.5.1310-1319.1996>

Rosqvist R, Skurnik M, Wolf-Watz H (1988) Increased virulence of *Yersinia pseudotuberculosis* by two independent mutations. Nature 334(6182):522-524. <https://doi.org/10.1038/334522a0>

Simon R, Priefer UB, Puhler A (1983) A broad host range mobilization system for *in vivo* genetic engineering: transposon mutagenesis in Gram negative bacteria. Nature Biotechnology 1(9):784-791

Zhu L, Zuo Y, Cui R, Fu P, Liu Y, Wang Z, He X, Yu D, Wei Z, Li S, Wang Y, Li C, Wang Y, Li DF, Liu SJ, Shen X (2026) Interkingdom sensing of fungal tyrosol promotes bacterial antifungal T6SS activity in the murine gut. Nat Microbiol 11(1):240-255. <https://doi.org/10.1038/s41564-025-02208-z>

Zuo Y, Li C, Yu D, Wang K, Liu Y, Wei Z, Yang Y, Wang Y, Shen X, Zhu L (2023) A Fur-regulated type VI secretion system contributes to oxidative stress resistance and virulence in *Yersinia pseudotuberculosis*. Stress Biol 3(1):2. <https://doi.org/10.1007/s44154-022-00081-y>
